# Effects of mental fatigue on biomechanical characteristics of lower extremities in patients with functional ankle instability during unanticipated side-step cutting

**DOI:** 10.3389/fphys.2023.1123201

**Published:** 2023-03-23

**Authors:** Lingyu Kong, Peng Wu, Xinwen Zhang, Lingyue Meng, Lintao Kong, Qiuxia Zhang, Jianzhong Shen

**Affiliations:** ^1^ Physical Education and Sports School, Soochow University, Suzhou, China; ^2^ School of Public Health, Suzhou Medical College of Soochow University, Suzhou, China; ^3^ Experimental Primary School, High Tech Zone Science and Technology City, Suzhou, China; ^4^ Rehabilitation Center, Shanghai Yongci Rehabilitation Hospital, Shanghai, China

**Keywords:** mental fatigue, functional ankle instability, side-step cutting, movement state, ankle stiffness

## Abstract

**Background:** Functional ankle instability (FAI) is the primary classification of ankle injuries. Competitive activities have complicated movements that can result in ankle re-injury among patients with FAI. Unanticipated movement state (MS) and mental fatigue (MF) could also happen in these activities, which may further increase their joint injury risk.

**Objective:** This study aimed to clarify the biomechanical characteristics difference of the lower extremity (LE) between the injured side and the uninjured side among patients with FAI when they perform unanticipated side-step cutting after MF.

**Methods:** Fifteen males with unilateral FAI participated in this study (age: 20.7 ± 1.3 years, height: 173.6 ± 4.4 cm, weight: 70.1 ± 5.0 kg). They used the injured side and the uninjured side of LE to complete anticipated and unanticipated side-step cutting before and after MF. The kinematic and kinetics data were evaluated using three-way ANOVA with repeated measures.

**Results:** During patients with FAI performed anticipated side-step cutting, the ankle stiffness of both sides showed no significant change after MF; During they performed unanticipated side-step cutting, their injured side presented significantly lower ankle stiffness after MF, while the uninjured side did not have such change. In addition, after MF, the injured side exhibited increased ankle inversion, knee valgus and LR, but the uninjured side did without these changes.

**Conclusion:** Influenced by MF, when patients with FAI use their injured side of LE to perform side-step cutting, this side LE has a higher risk of musculoskeletal injuries such as lateral ankle sprains and anterior cruciate ligament injury. The ankle stiffness of the injured side will be further reduced when patients with FAI perform unanticipated side-step cutting, which increases ankle instability and the risk of re-injury.

## 1 Introduction

Lateral ankle sprain is the most common type of sports injury in the lower extremity (LE), with an incidence rate of approximately 7.3%; it is mostly caused by abrupt excessive ankle inversion (Roos et al., 2017). If the injured ankle is not properly treated after the first sprain, it will continue to be negatively affected by chronic pain and local edema. Patients might be unable to control the injured ankle sufficiently, increasing its re-injury risk and eventually leading to functional ankle instability (FAI) (Cao et al., 2019).

FAI is the primary classification of ankle instability in clinical, and it can be attributed to the loss of proprioception and neuromuscular deficits in the injured ankle (Steib et al., 2013). FAI is typically accompanied by abnormal joint sensation and loss of normal motion control of the ankle (Freeman et al., 1965; Marinho et al., 2017). The abnormal joint sensation usually occurs in patients with FAI (Freeman et al., 1965; Arnold et al., 2009; Lysdal et al., 2022), which refers to frequently unconscious episodes of hyper-inversion and subsequent “giving way” of the ankle likely to occur (Tropp, 2002; Takeda et al., 2021). Such abnormal ankle motion may aggravate the risk of ankle re-injury in patients with FAI during sports. Side-step cutting is one of the complex movements commonly used in competitive activities, and its main objective is to dodge the defensive player through sudden direction changes while running (Besier et al., 2001). This movement usually generates an impact on the ankle joint that is three times more than one’s body weight, and the generated instantaneous pressure is largely borne by the LE. Recent studies have shown that patients with ankle instability exhibit significant biomechanical differences in side-step cutting performance compared with healthy individuals, especially the significantly greater ankle internal rotation that may result in ankle re-injury (Simpson et al., 2020a; Simpson et al., 2020b).

When humans perform movements, they need to fully account for the external environment, and follow-up actions will be preplanned under the current movement state (MS) (Dey and Schilling, 2022). MS in competitive sports is constantly changing. Participants do not know the direction or route of action in advance and need to make immediate adjustments based on the instant feedback from the sensory system (Besier et al., 2001). Sudden unanticipated disturbances can evoke psychological and physiological responses to such stimulus, namely, the startle reflex. The main function of the startle reflex is to help the body avoid external stimulation and increase the sympathetic nervous system activity to prepare for subsequent actions (Yeomans et al., 2002). But at the same time, because the startle reflex is an autonomous defensive reflex and is not controlled by the will, its appearance is likely to lead to the deformation of the standard action. In unanticipated MS, the human body cannot quickly and accurately make corresponding postural adjustments like anticipated MS, resulting in involuntary changes in neuromuscular activities. Some scholars pointed out that unanticipated side-step cutting increases the knee valgus angle and the ligament injury risk (Brown et al., 2014). Patients with FAI have a diminished capacity to use their injured side of LE to maintain balance. They cannot adapt to the external environment and adjust their posture in time (Kazemi et al., 2017). They might be more likely to suffer from injury when performing unanticipated side-step cutting. Analyzing the biomechanical characteristics of these patients who perform unanticipated side-step cutting can provide a theoretical basis and references for preventing sports injury.

Patients with ankle instability experience difficulty maintaining postural balance after prolonged and intensive exercise due to proprioceptive deficits and weak ankle motion control (Gribble et al., 2007), making them more at risk of excessive ankle torsion. Both mental fatigue (MF) and muscle fatigue caused by long-term exercise could reduce sports performance quality. But different from muscle fatigue, MF is a psychobiological state caused by prolonged periods of demanding cognitive activity (Van Cutsem et al., 2017; Le Mansec et al., 2018; Meeusen et al., 2021), emphasizing the difficulty of CNS signal integration and the increased cognitive burden (Li et al., 2015). Previous studies have revealed that individuals are unable to concentrate and maintain efficiency in working after MF (Ream and Richardson, 1996) and also have difficulty changing coping strategies in the face of the external environment or in performing movement tasks (Lorist et al., 2005; Pageaux and Lepers, 2018). After moderate-to low-intensity aerobic exercise, jeopardized behavioural and cognitive control induced by MF disables the general muscle contraction even though muscles are without fatigue (Olson et al., 2016). Although both MF and MS have been proven to be potential factors affecting sports safety, most studies only analyzed the movement performance of patients with FAI when they complete the anticipated side-step cutting (Dayakidis and Boudolos, 2006; Suda and Sacco, 2011). A few studies have analyzed the biomechanical performance of these patients while they perform the unanticipated step-cutting task (Kim et al., 2021a; Kim et al., 2021b), but scholars have not further considered the possible effects of MF in this state.

The current study is aimed to clarify the biomechanical characteristics of the LEs of patients with FAI during anticipated or unanticipated side-step cutting before and after MF. We hypothesized the following: 1. The patients with FAI show different kinematic and kinetic characteristics between the injured and the uninjured sides during side-step cutting; 2. The appearance of MF or unanticipated movement affects the kinematic and kinetic characteristics of the injured side during side-step cutting; 3. Influenced by MF, the injured side could show obvious biomechanical characteristics changes during these patients perform unanticipated side-step cutting in comparison with the uninjured side.

## 2 Materials and methods

### 2.1 Participants

Fifteen males with unilateral FAI (age: 20.7 ± 1.3 years, height: 173.6 ± 4.4 cm, weight: 70.1 ± 5.0 kg) who frequently participated in sports (i.e., at least three times a week) and had good side-step cutting skills, were recruited for this study. The dominant LE of all the participants was right determined by kicking a ball; it was also the injured side. The inclusion criteria for patients with FAI were as follows. (1) The unilateral ankle had suffered at least one sprain in the past year, and the patient experienced a feeling of weakness or instability. (2) The score in the Cumberland Ankle Instability Tool was below 24 (Donahue et al., 2011); (3) The patient had no critical LE injury history, including fractures or serious orthopedic injury (Wu et al., 2022), except for ankle sprain; (4) The anterior drawer test and the talar tilt test were negative (Kaminski et al., 1999); (5) Only one LE side suffered from FAI. The exclusion criteria were as follows: (1) Bilateral ankle sprains (Wang et al., 2022); (2) Acute pathological symptoms of LE; (3) History of previous surgeries in the LE (Kweon et al., 2022); (4) History of equilibrium and balance control disorder (Wu et al., 2022); (5) Had congenital feet, ankles, knees, pelvis and spine deformities. This study was approved by Ethics Committee of Soochow University, and all the participants had written consent forms before the experiment.

### 2.2 Side-step cutting

The design of the side-step cutting task followed that of previous research (Kim et al., 2014; Liew et al., 2021). In summary, a minimum of 8 m run-up distance was provided to participants to reach the desired speed (4.0 ± 0.5 m/s). Participants stepped their foot on the fixed position of the force platform (90 cm × 60 cm × 10 cm) in the mode of rear foot landing. Then they quickly ran away from the force platform towards the cutting direction. When participants completed the left-direction side-step cutting, they must complete the cutting task by the right side of LE. On the contrary, they needed to use the left side of LE during the cutting direction is right. All participants were required to run at least three steps before decelerating and stopping.

**FIGURE 1:**
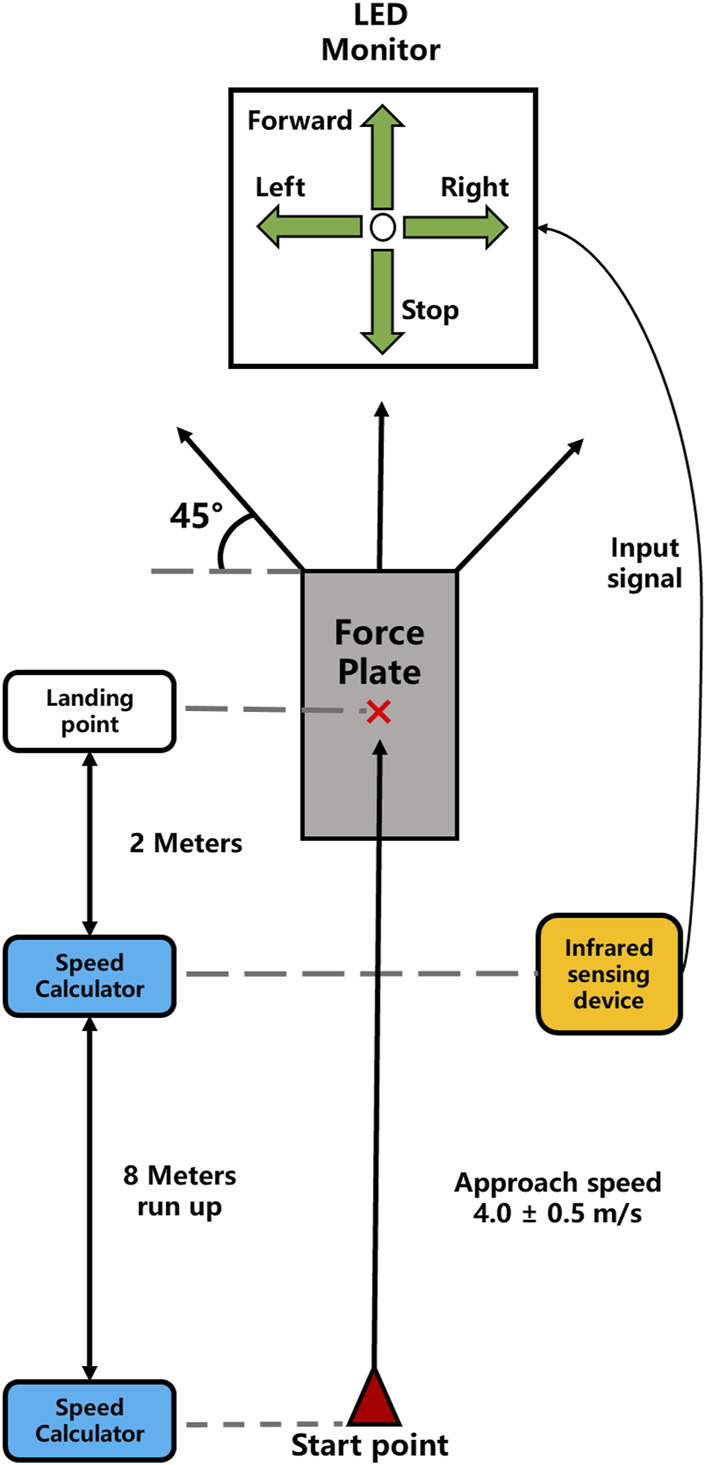
Layout of experimental site.

Side-step cuttings were performed under anticipated and unanticipated states in sequence. A light-emitting diode (LED) monitor with four green bulbs in the arrow shape was set in behind the force platform to give the side-step cutting direction order. The left (right) arrow represented participants should perform maximum-effort side-step cutting to the left (right) 45° direction. The upper arrow representative continued to run forward, while the lower arrow representative emergently stopped. For the anticipated state, the bulb with the left (or right) arrow has lit before participants run up. For the unanticipated state, the LED monitor was connected to the infrared sensing device. Only when the participants passed the infrared sensing device was one of the four arrow bulbs applied through a computer program randomly lit. The layout of experimental site is shown in Figure 1.

### 2.3 Induction and assessment of MF

The Stroop task has been proven effective in inducing MF (Mangin et al., 2022). In the current study, a 45-min Stroop task included four words in Chinese (red, yellow, blue, and green) that were displayed randomly in one of the four colors. The task was presented in an event-related design. It comprised 675 trials that included 225 congruent trials (the meaning of the color matched the color) and 450 incongruent trials (the meaning of the color did not match the color). Each trial began with a 500 ms fixation cross, followed by the stimulus presented for 2 s and then the blank for 1.5 s. The subjects were required to press one of the four keys to indicate the color of the ink while disregarding the meaning of the color word.

As a complex probe of attention that combines both visual selectivity and motor response, letter cancellation task (LCT) can reflect the participants’ changes in attention, attention span, and vigilance (Casagrande et al., 1997; Casagrande et al., 1999; Pradhan et al., 2018), and can be used in neurological status assessment (Geldmacher, 1998). In this study, the degree of participants’ MF before and after receiving the 45-min Stroop task were assessed by LCT. Participants were required to search for and mark target letters as quickly and accurately as possible (Casagrande et al., 1997). Target letters were randomly placed within a 20 × 53 matrix of capital letters (font: Time New Roman, size: 12) printed on an A4-size paper. Participants were ordered to complete LCT as soon as possible. Different matrices of capital letters were used to analyze the changes in the level of MF in each participant before and after they received the Stroop task. Whether the changes in the performance of LCT in participants were significantly worsened were used to determine their appearance of MF.

### 2.4 Experimental procedure

All the participants were asked to refrain from strenuous exercises 24 h before the formal test. Before data collection, they performed a warm-up exercise that comprised of a 5-min jogging on a treadmill at their preferred speed (Zebris FDM-T, Germany).

All participants first completed three successful trials of side-step cutting movement under both anticipated and unanticipated conditions, performed the Stroop task to induce MF, and then repeated three successful trials of the same side-step cutting movement under the two testing conditions. Data were collected and recorded during each trial. A successful trial was defined as a run-up speed within 4.0 ± 0.5 m/s. Side-step cutting should realize the correct footfalls and land on the point. The cutting angle must be within 45° ± 3°. The mean value of three trials was used for comparative analysis. Finally, the successful 45° side-step cutting of the uninjured side and injured side under different MSs before and after MF was analyzed and compared. Running forward and emergent stops were used as interference items under unanticipated MS, and their trial data were not considered in the subsequent analysis and comparison.

### 2.5 Data processing

Kinematic data were captured using a motion analysis system that comprised eight infrared cameras (Vicon Motion Analysis, United Kingdom) by tracking 16 infrared reflective balls (reflective markers) with a diameter of 14 mm at 100 Hz. The infrared reflective balls were stick to participants’ corresponding parts following the scheme suggested by the Plug-in Gait model. Kinetic data were capture using a 3D force plate (KISTLER, Switzerland) at 1,000 Hz, which was synchronized with motion analysis system. Kinematic and kinetic data were firstly processed by Vicon Nexus 2.1.2. Both kinematic and kinetic data were then imported to Visual3D (Version 6, C-Motion, Inc, United States) for further processing. The following data were analyzed: peak joint angles on the sagittal and frontal planes (Kim et al., 2014); ground-reaction force (GRF), including peak vertical GRF (vGRF), peak medial GRF (mGRF), peak horizontal GRF (hGRF); time-to-peak ground reaction force (T_GRF), including time-to-peak vertical GRF (T_vGRF), time-to-peak medial GRF (T_mGRF), time-to-peak horizontal GRF (T_hGRF), and stance duration; ankle stiffness and loading rate (LR). GRF data were standardized by each participant’s body weight (BW).

Working efficiency can comprehensively evaluate the performance of participants when completing LCT (Yang, 1989). We used working efficiency to analyze the fatigue degree of the central nervous system (CNS) to determine whether MF was successfully induced. Working efficiency was calculated using Equation 1.

$$A=\frac{\left(c-w\right)}{\left(c+o\right)}E=100*A/T$$ (1)

A denotes the accuracy of cancellation, c represents the number of cancelled symbols, w represents the number of wrongly cancelled symbols, o represents the number of missed cancelled symbols, T is the time taken to cancel symbols, and E indicates working efficiency.

LR can be raised due to load accumulation caused by abnormal movement patterns, which are closely associated with injury. LR was calculated using Equation 2.

LR=vGRF/T_vGRF (2)

Ankle joint stiffness is the ratio of the change of ankle joint moment and ankle joint angular displacement from the moment of touchdown to the moment of maximum ankle dorsiflexion (Kim et al., 2019). In the current study, we used the ankle stiffness of different LEs to quantify the interaction between MF and MS. Ankle stiffness was normalized to each participant’s BW. Ankle stiffness was calculated using Equation 3.

$$K_{\mathrm{ankle}}=\Delta M_{\mathrm{ankle}}/\Delta \theta_{\mathrm{ankle}}$$ (3)

ΔM is defined as the change in ankle moment, and Δθ represents the angular displacement in ankle dorsiflexion (Hamill et al., 2014).

### 2.6 Statistical analysis

Data were expressed as mean and standard deviation (SD). SPSS 26.0 software (SPSS Inc, Chicago, IL, United States) was used for statistical analysis. Normality tests were conducted *via* the Kolmogorov–Smirnov test. Matched sample *t*-test was used to assess the working efficiency of the participants before and after the Stroop task. A three-way (2 LE × 2 MF × 2 MS) analysis of variance with repeated measures was performed for kinematic and kinetic variables. Interaction effects will be investigated prior. Main effects were considered only if non-significant interaction effects were found. In case a significant interaction was detected, simple effects analysis was performed (Keppel et al., 1992). Statistical significance was set at 0.05 for all variables.

## 3 Results

**FIGURE 2:**
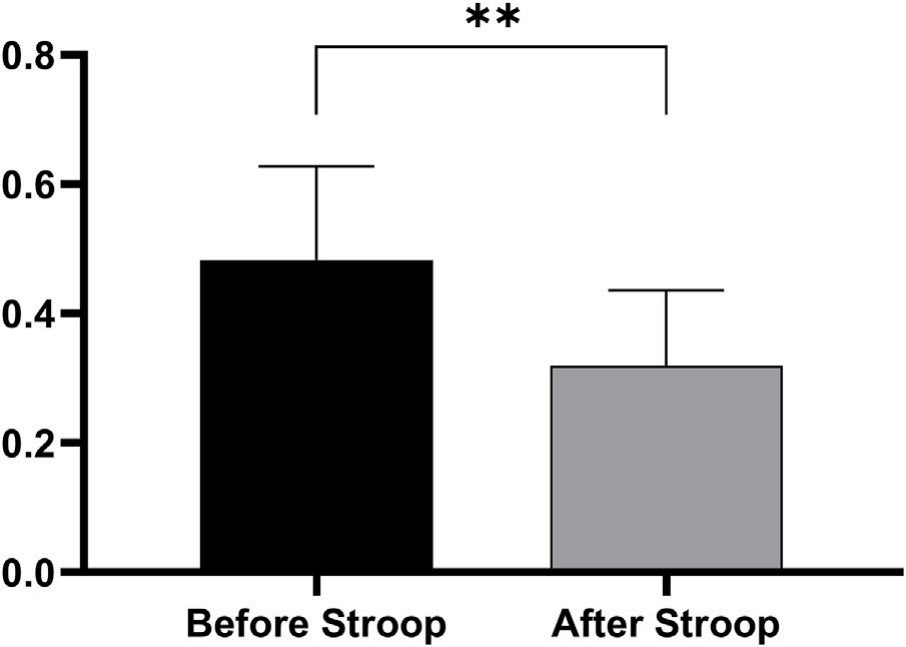
Changes in work efficiency of LCT in participants before and after Stroop task.

Figure 2 displays the results of the change in the working efficiency of LCT before and after the Stroop task. All the participants demonstrated a remarkable decrease in work efficiency after the Stroop task (*t* = 3.097, *p* = 0.008), indicating that MF was successfully induced.

Table 1 presents the results in comparison of the peak joint angles between the injured side and the uninjured side. LE (*F* = 6.704, *p* = 0.015, η^2^ = 0.193), MF (*F* = 11.512, *p* = 0.002, η^2^ = 0.291), and MS (*F* = 7.100, *p* = 0.013, η^2^ = 0.202) showed significant main effects on ankle dorsiflexion. LE × MF exhibited a significant interaction effect on ankle inversion (*F* = 4.624, *p* = 0.040, η^2^ = 0.142). The simple effect analysis showed that the injured side had significantly increased ankle inversion after MF (*p* = 0.021); However, the uninjured side did not have a similar change (*p* = 0.566). MS showed a significant main effect on knee flexion (*F* = 13.486, *p* = 0.001, η^2^ = 0.325). LE (*F* = 4.475, *p* = 0.043, η^2^ = 0.138), and MF (*F* = 5.094, *p* = 0.032, η^2^ = 0.154) showed significant main effects on knee valgus. LE × MF showed a significant interaction effect on knee valgus (*F* = 4.516, *p* = 0.043, η^2^ = 0.139). The simple effect analysis showed that the injured side had significantly increased knee valgus after MF (*p* = 0.003), while the uninjured side did not exhibit such a change (*p* = 0.926). MS exhibited a significant main effect on hip abduction (*F* = 5.607, *p* = 0.025, η^2^ = 0.167).

**TABLE 1:** Maximum Angles of the hip, knee, and ankle joints during side-step cutting.

Variables (+/−)	Injured side		Uninjured side		Main effect			Interaction effect			
	Anticipated movement	Unanticipated movement	Anticipated movement	Unanticipated movement	LE	MF	MS	LE × MF	LE × MS	MF × MS	LE × MF × MS
**Ankle dorsiflexion/plantarflexion (degree)**											
Before MF	18.8 (5.6)	19.2 (5.7)	20.2 (5.9)	23.5 (6.1)	**0.015** ^*^	**0.002** ^**^	**0.013** ^*^	0.971	0.574	0.424	0.318
After MF	20.1 (4.8)	23.7 (4.9)	23.3 (4.3)	26.2 (4.0)							
**Ankle eversion/inversion (degree)**											
Before MF	−10.5 (5.8)	−10.3 (3.1)	−10.2 (6.2)	−10.6 (4.6)	0.118	0.202	0.296	**0.040** ^*^	0.608	0.300	0.358
After MF	−11.6 (5.9)	−15.1 (7.4)	−9.3 (5.0)	−10.0 (4.2)							
**Knee flexion/extension (degree)**											
Before MF	45.7 (5.1)	51.9 (6.4)	47.6 (8.0)	51.5 (6.4)	0.681	0.971	**0.001** ^**^	0.864	0.984	0.975	0.480
After MF	47.1 (7.5)	51.1 (5.3)	46.2 (11.2)	52.5 (8.8)							
**Knee valgus/varus (degree)**											
Before MF	3.7 (5.0)	4.5 (3.1)	3.6 (6.2)	4.3 (4.1)	**0.043** ^*^	**0.032** ^*^	0.057	**0.043** ^*^	0.333	0.434	0.417
After MF	6.1 (4.6)	9.7 (4.5)	3.7 (5.0)	4.4 (5.2)							
**Hip flexion/extension (degree)**											
Before MF	42.9 (13.7)	49.2 (14.4)	48.6 (9.6)	50.3 (9.3)	0.210	0.973	0.098	0.971	0.811	0.684	0.341
After MF	45.5 (11.2)	46.6 (15.3)	47.4 (9.9)	51.2 (4.9)							
**Hip abduction/adduction (degree)**											
Before MF	4.3 (3.2)	6.6 (7.6)	4.5 (7.2)	8.1 (4.8)	0.803	0.351	**0.025** ^*^	0.648	0.146	0.877	0.538
After MF	4.0 (6.0)	5.7 (4.6)	6.8 (6.1)	7.0 (5.7)							

“*” means significance (*p*< 0.05), “**” means very significance (*p*< 0.01). Abbreviations: LE: lower extremity, MF: mental fatigue, MS: movement state.

Bold value is the appearance of statistical significance.

Table 2 presents the results in comparison of GRF, T_GRF and stance duration between the injured side and the uninjured side. MS showed significant main effects on vGRF (*F* = 10.313, *p* = 0.003, η^2^ = 0.269), mGRF (*F* = 55.882, *p* < 0.001, η^2^ = 0.666), and T_vGRF (*F* = 10.485, *p* = 0.003, η^2^ = 0.272). LE presented a significant main effect on hGRF (*F* = 11.198, *p* = 0.002, η^2^ = 0.286). No interaction effects were observed in this table.

**TABLE 2:** Peak ground reaction force, time to peak ground reaction force, and stance duration during side-step cutting.

Variables	Injured side		Uninjured side		Main effect			Interaction effect			
	Anticipated movement	Unanticipated movement	Anticipated movement	Unanticipated movement	LE	MF	MS	LE × MF	LE × MS	MF × MS	LE × MF × MS
**vGRF (BW)**											
Before MF	3.20 (0.75)	2.76 (0.79)	3.38 (0.65)	2.63 (0.71)	0.463	0.593	**0.003** ^**^	0.372	0.436	0.229	0.741
After MF	3.27 (0.51)	3.09 (0.91)	3.09 (0.94)	2.81 (0.75)							
**mGRF (BW)**											
Before MF	0.96 (0.29)	0.71 (0.17)	0.83 (0.21)	0.73 (0.18)	0.892	0.330	**0.000** ^**^	0.136	0.060	0.476	0.589
After MF	0.88 (0.14)	0.62 (0.16)	0.90 (0.10)	0.70 (0.22)							
**hGRF (BW)**											
Before MF	1.06 (0.26)	1.18 (0.22)	1.21 (0.21)	1.23 (0.20)	**0.002** ^**^	0.836	0.509	0.593	0.474	0.418	0.784
After MF	1.08 (0.25)	1.10 (0.29)	1.25 (0.23)	1.21 (0.26)							
**T_vGRF (s)**											
Before MF	0.049 (0.011)	0.043 (0.015)	0.055 (0.011)	0.042 (0.011)	0.375	0.321	**0.003** ^*^	0.976	0.148	0.196	0.906
After MF	0.044 (0.008)	0.043 (0.014)	0.049 (0.017)	0.043 (0.012)							
**T_mGRF (s)**											
Before MF	0.226 (0.053)	0.212 (0.077)	0.254 (0.054)	0.208 (0.057)	0.391	0.092	0.586	0.977	0.162	0.079	0.980
After MF	0.223 (0.038)	0.256 (0.083)	0.249 (0.089)	0.252 (0.070)							
**T_hGRF (s)**											
Before MF	0.095 (0.023)	0.101 (0.037)	0.105 (0.021)	0.097 (0.027)	0.812	0.089	0.563	0.775	0.397	0.751	0.708
After MF	0.110 (0.020)	0.107 (0.037)	0.112 (0.041)	0.105 (0.029)							
**Stance duration (s)**											
Before MF	0.379 (0.087)	0.387 (0.139)	0.392 (0.083)	0.378 (0.102)	0.907	0.096	0.502	0.987	0.610	0.495	0.941
After MF	0.399 (0.070)	0.434 (0.140)	0.409 (0.144)	0.429 (0.119)							

“*” means significance (*p*< 0.05), “**” means very significance (*p*< 0.01). Abbreviations: LE: lower extremity, MF: mental fatigue, MS: movement state.

Bold value is the appearance of statistical significance.

**FIGURE 3:**
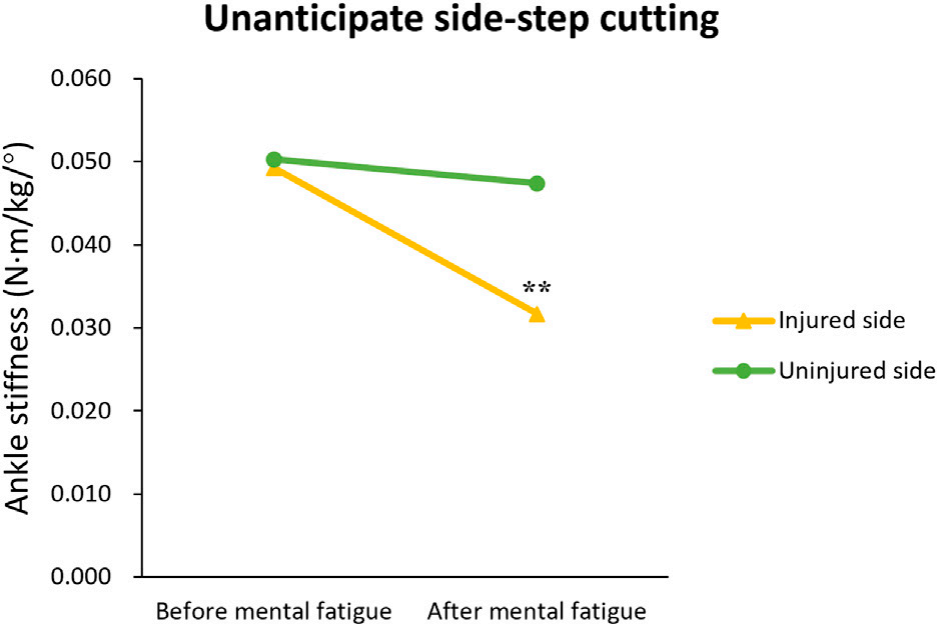
Change trend in ankle stiffness among patients with functional ankle instability using the injured and the uninjured sides lower extremities perform unanticipated side-step cutting before and after mental fatigue. “**” means a very significant statistical difference compared with before mental fatigue (*p* < 0.01).

Table 3 provides the results in comparison of ankle stiffness and loading rate between the injured and the uninjured side. LE (*F* = 7.523, *p* = 0.011, η^2^ = 0.212), MF (*F* = 17.738, *p* < 0.001, η^2^ = 0.388) and MS (*F* = 10.177, *p* = 0.003, η^2^ = 0.267) have significant main effects on ankle stiffness; LE × MS (*F* = 4.659, *p* = 0.040, η^2^ = 0.143), and LE × MF × MS (*F* = 4.373, *p* = 0.046, η^2^ = 0.135) have significant interaction effects on ankle stiffness. The simple effect analysis of LE × MF × MS showed that during anticipated cutting, both sides showed no significant change in ankle stiffness after MF (*p* = 0.752; *p* = 0.399); During unanticipated cutting, the injured side presented significantly decreased ankle stiffness after MF (*p <* 0.001), whereas the uninjured side did not exhibit such change (*p* = 0.963) (Figure 3).

**TABLE 3:** Ankle stiffness and lower extremities loading rate during side-step cutting.

Variables	Injured side		Uninjured side		Main effect			Interaction effect			
	Anticipated movement	Unanticipated movement	Anticipated movement	Unanticipated movement	LE	MF	MS	LE × MF	LE × MS	MF × MS	LE × MF × MS
K_ankle_ (N·m/kg/°)											
Before MF	0.051 (0.012)	0.049 (0.009)	0.054 (0.009)	0.050 (0.007)	**0.011** ^*^	**0.000** ^**^	**0.003** ^**^	0.139	**0.040** ^*^	0.173	**0.046** ^*^
After MF	0.049 (0.011)	0.032 (0.010)	0.048 (0.011)	0.047 (0.011)							
LR (BW/s)											
Before MF	65.08 (4.33)	65.96 (5.37)	61.99 (6.00)	63.32 (5.35)	**0.001** ^**^	0.000**	0.834	**0.001** ^**^	0.163	0.314	0.372
After MF	74.74 (5.87)	72.05 (6.75)	64.33 (6.13)	65.44 (4.69)							

“*” means significance (*p*< 0.05), “**” means very significance (*p*< 0.01). Abbreviations: LE: lower extremity, MF: mental fatigue, MS: movement state.

Bold value is the appearance of statistical significance.

LE (*F* = 14.588, *p* = 0.001, η^2^ = 0.343) and MF (*F* = 43.942, *p* < 0.001, η^2^ = 0.611) showed significant main effects on LR. LE × MF exhibited a significant interaction effect on LR (*F* = 13.769, *p* = 0.001, η^2^ = 0.330). The simple effect analysis showed that the injured side had higher LR after MF (*p* < 0.001), but the uninjured side did not show changes (*p* = 0.069).

## 4 Discussion

In this study, we found the biomechanical characteristics difference of the lower extremity (LE) between the injured side and the uninjured side among patients with FAI when they performed side-step cutting. This finding approved our hypothesis 1; Mainly MF but not MS would affect these patients’ biomechanical characteristics of LE. This finding partly approved our hypothesis 2; Consistent with our hypothesis 3, we observed that during unanticipated side-step cutting, the ankle stiffness of the injured side decreased significantly after MF. Since we observed interaction effects, we mainly discussed these findings next.

Consistent with the previous study, MF induced by the Stroop test negatively affected the kinematic performance among elite sporters (Veness et al., 2017); our results showed that MF induced by the Stroop test influenced the normal motion control of patients with FAI in their ankle and knee in the front plane of the injured side. The side-step cutting movement has high requirements for the non-sagittal movement of the LE when landing (Zhou et al., 2021). The function of muscles in controlling the ankle in patients with FAI is impaired (Delahunt et al., 2006), and Koshino et al. (2016) found that the ankle inversion angle of patients with ankle instability was significantly higher than that of healthy individuals when completing side-step cutting. Our results are consistent with them and showed that the injured side had increased ankle inversion compared to the uninjured side after MF. If the ankle inversion of patients with ankle instability is reduced while performing 45° side-step cutting, representing they take prudent and conservative sports strategies to avoid ankle injury (Son et al., 2017; Fuerst et al., 2018). For patients with FAI, they must exert more energy to make their CNS consciously control ankle stability. Reduced CNS control caused by MF inhibits such protection and finally increases ankle inversion. Similarly, Pageaux et al. (2014) found that 30 min of mental exertion involving response inhibition reduces subsequent self-paced endurance performance, negatively affecting the normal motion control of LE. Increased ankle inversion indicates that the lateral ankle musculature of patients with FAI is unable to control frontal-plane motion eccentrically when the lateral ankle is loaded during ground contact, which leads to the ankle complex giving way to excessive inversion and exposing the injured side to an increased risk of recurrent lateral ankle sprain during side-step cutting (Simpson et al., 2020b).

Our results also showed that the injured side had increased knee valgus compared with the uninjured side after MF. This result is in line with the research view of David et al. (2017), that is, the increased knee valgus will better meet the needs of people who suffer from ankle injuries. In the current study, all patients with FAI were ordered to adopt the rear foot landing mode. vGRF of the rear foot landing is 3.4 times that of the front foot landing mode (Kovács et al., 1999), and the knee joint turn outward can better absorb vGRF (Mizuno et al., 2009) to reduce the ankle needs to bear when performing movements. The increased knee valgus of the injured side proved that the appearance of MF affected and adjusted the GRF distribution of LE, reducing the ankle burden. However, Son et al. (2017) have found that although elastic ankle protection can reduce the energy absorption of the ankle, which protects the damaged ankle joint, the negative impact still has on the knee joint. Most patients with ankle instability reported more symptomatology in the knee and worse knee joint health than healthy individuals (Kosik et al., 2020). Increased knee valgus can induce anterior cruciate ligament injury during side-step cutting (McLean et al., 2005; David et al., 2017). Hence, influenced by MF, patients with FAI rely more on changing knee motion to cushion the GRF of side-step cutting, but this further aggravates the risk of knee joint injury.

In this study, the injured side showed higher LR after MF, consistent with Tajdini et al. (2022) that patients with ankle instability exhibited a greater inter-limb asymmetry of LR, and the LR of their injured side was higher than healthy individuals during walking. As we found in kinematics, the FAI population after MF cannot effectively control the ankle inversion angle. Greater ankle movement control can decrease LR (Decker et al., 2002), reducing the impact of stress on soft tissues during landing. The impaired ability in injured ankle motion control of patients with FAI might be the main reason for the high LR. Besides, some scholars (Le Mansec et al., 2018) pointed out that MF directly affects the depth and speed of visual processing before attention. These changes negatively affect the precision and integrity of automatic visual processing, influencing the subsequent concentrated attention stage processing and ultimately leading to error task execution (Rozand et al., 2015; Smith et al., 2016). In side-step cutting, the human body must brake to reduce the forward speed when touching the ground first and then transfer part of the forward speed to the side speed by pushing the ground in the opposite direction of the side cut, accelerating the push to achieve the purpose of a side cut. All the actions mentioned above should be completed quickly. Due to the damaged muscle spindle and around receptors of the injured ankle, nerve signal transmission speed from the joint to the CNS was directly impeded. The appearance of MF will further negatively influence the signal process of CNS, and sensory information will not be processed in time. These patients adopt an uncomplicated buffering strategy to complete side-step cutting, which requires less time, and posture preparation process of the injured side, but soft tissues or muscles of the injured side of LE are not fully mobilized to cushion the load during side-step cutting, making raised LR, which will cause stress fracture and plantar fasciitis (Venesky et al., 2006).

When the posture of the human body changes, the adjustment of LE stiffness is the first thing to start (Liu et al., 2006). If joint stiffness cannot effectively adjust the impact and influence generated by MS, it will inevitably reduce the quality of movement performance, such as the deformation of LE movements during landing (Flanagan and Harrison, 2007; Balasundaram and Rajan, 2018), leading to musculoskeletal injury. During the anticipated MS, humans can prepare enough to adjust the physical characteristics of soft tissues by actively activating muscles, the neuromuscular system will adjust muscle tuning according to the MS (Nigg and Wakeling, 2001), and the stiffness and motion of joints will also be adjusted accordingly to activate the joint soft tissue and reduce the risk of injury (Nigg and Liu, 1999). Increased ankle stiffness can be used to stabilize the body and prevent excessive joint motion (Brughelli and Cronin, 2008; Li et al., 2021). In this study, MF had no significant influence on ankle stiffness of the injured side when patients with FAI performed anticipated side-step cutting. Consistent with our results, Simpson et al. (2020a) found that less lateral center of pressure progression and increased tibialis anterior activation were observed in patients with ankle instability, reflecting a protective movement strategy during anticipated side-cutting to avoid recurrent injury. Therefore, when patients with FAI use their injured side to perform the anticipated side-step cutting, the appearance of MF could not result in an obviously negative influence on the ankle joint.

However, the amount of time for participants to make appropriate postural adjustments before performing the unanticipated cutting task is smaller than the anticipated state (Besier et al., 2001), and their movement plan must be immediately adjusted in the CNS based on current MS. In this process, the thinking decision is dominant in their brain, but their movement control is inevitably weakened. The appearance of MF will further delay the thinking decision process, leading to abnormal action control (Pageaux et al., 2014). We found that, influenced by MF, the injured side had significantly decreased ankle stiffness when these patients performed unanticipated side-step cutting. This finding is consistent with Kim et al. (2019) that the ankle stiffness of patients with ankle instability is lower than that of healthy individuals. Brown et al. (2022) revealed that the decreased ankle stiffness of the injured side indicates its diminished ability to respond to movement loading. A compliant joint contributes more to the attenuation of the joint load than a stiffer joint (Hamil et al., 2014), and such altered neuromechanics in patients with ankle instability means redistributing energy absorption from the distal (ankle) to the proximal (knee and hip) joints (Kim et al., 2019). The ankle stiffness change might be a protection strategy used by patients with FAI (Sarvestan et al., 2021) to modulate impact forces during this task. But due to side-step cutting being a complex and intense exercise, the decreased ankle stiffness also means their ankle stability will be weaker in this task. A previous study revealed that patients with ankle instability demonstrated alterations in landing/cutting movement strategies. These patients have a higher susceptibility to foot placement for lateral ankle sprains (Kim et al., 2019). Consequently, influenced by MF, when patients with FAI use their injured side to perform unanticipated side-step cutting, their injured ankle easily suffers from re-injury.

## 5 Clinical implications

This study demonstrated that compared with MS, MF is more likely to lead to patients with FAI having biomechanical characteristics changes in the injured side of LE. Proper physical exercise is very important to improve the lower limb joint control of this group of people. Balance training can improve the neuromuscular control ability of people with FAI when completing dynamic tasks, thus improving test performance (Wu et al., 2022). The findings of kinematics suggest that after MF, patients with FAI have control obstacles in sagittal control of ankle and knee joints. Progressive hop-to-stability balance (PHSB) training is a balance training method that emphasizes dynamic stability in predictable or unpredictable jump direction changes, take-off and landing plane and dynamic touch tasks. Previous studies have applied the PHSB training method proposed by McKeon et al. (2008) to train patients with ankle instability and have suggested that PHSB training is of great help in improving the posture control of people with ankle instability (McKeon et al., 2009; Anguish and Sandrey, 2018). Ardakani et al. (2019) further found that after patients with ankle instability received a 6-week hop-stabilization training program, they had better control of frontal-plane joint angles at the ankle and knee, which reduced the joint injury risk. This finding suggests that PHSB might greatly improve the kinematic performance of patients with FAI during side-step cutting and minimize the influence of MF.

Besides, influenced by MF, when patients with FAI use their injured side to perform side-step cutting, this side has higher LR than their uninjured side. Since this population relies heavily on the feedforward function to adjust posture (Delahunt et al., 2006), treatment personnel can increase their feedforward function training to maintain the posture control ability of these patients and prevent potential injuries induced by abnormal kinetic changes after MF. Some scholars have reported that gait retraining programs that utilize biofeedback can reduce high LR caused by abnormal movement patterns (Phan et al., 2017), which may be an effective training method for these patients to reduce the higher LR of the injured side. In addition, when performing unanticipated side-step cutting, the changes in ankle stiffness of the injured side ankle after MF may induce injury risk to the injured ankle. Treatment personnel should delay the emergence of MF in patients with FAI to avoid the possibility of fatigue injury to these patients’ ankles. Some scholars (Chen and Zhang, 2021) have proposed that moderate but not excessive exercise can improve the level of brain metabolism and balance the secretion of neurotransmitters, improving cognition. Designing targeted exercise treatment plans for this population is worth exploring in the future.

## 6 Limitation

This study still has some limitations. Although this study tries to restore the performance of patients with FAI when they complete the side-step cutting in a natural sports environment and analyze their biomechanical characteristics, the number of samples is still small, which may lead to a decline in the effectiveness of some results. Besides, ankle muscles play a vital role in postural stability. Since electromyography was not used in this study, the activation of those muscles is still not apparent during the experimental process, and relevant indexes of electromyography can be considered in future research.

## 7 Conclusion

In conclusion, MF and MS affect the LE biomechanical characteristics of patients with FAI during side-step cutting. Influenced by MF, patients with FAI will adopt protective strategies by increasing knee valgus to buffer GRF when using their injured side of LE to complete side-step cutting. However, an increased load and a higher risk of musculoskeletal injuries such as lateral ankle sprains and anterior cruciate ligament injuries remain on this side LE. In particular, when their brain is fatigued, and patients with FAI perform unanticipated side-step cutting using the injured side of LE, ankle stiffness considerably decreases, which enhances the possibility of bone and soft tissue damage. To improve the safety of the joints of patients with FAI during competitive sports, we suggest these patients should avoid performing unanticipated side cutting after MF. Athletic trainers and treatment personnel need to take appropriate treatment to improve these patients’ cognition to delay the occurrence of MF.

## Data Availability

The raw data supporting the conclusion of this article will be made available by the authors, without undue reservation.
